# Where Are We and Where to Next?—The Future of Perianal Crohn’s Disease Management

**DOI:** 10.3390/jcm12196379

**Published:** 2023-10-06

**Authors:** Sulak Anandabaskaran, Luke Hanna, Nusrat Iqbal, Laura Constable, Phil Tozer, Ailsa Hart

**Affiliations:** 1 Department of Metabolism, Digestion and Reproduction, Faculty of Medicine, Hammersmith Campus, Imperial College London, London W12 0NN, UK; 2 Robin Phillip’s Fistula Research Unit, St Mark’s Hospital and Academic Institute, London HA1 3UJ, UK; 3 Faculty of Medicine, St Vincent’s Clinical School, University of New South Wales, 390 Victoria Street, Darlinghurst, NSW 2010, Australia; 4 Department of Surgery and Cancer, South Kensington Campus, Imperial College London, London SW7 2BX, UK

**Keywords:** inflammatory bowel disease, perianal Crohn’s Disease, management

## Abstract

Perianal fistulizing Crohn’s Disease (pCD) affects about 25% of patients with Crohn’s Disease (CD). It remains a difficult entity to manage with a therapeutic ceiling of treatment success despite improving medical and surgical management. The refractory nature of the disease calls for an imminent need to better understand its immunopathogenesis and classification to better streamline our treatment options. In this article, we overview the current state of pCD management and discuss where the future of its management may lie.

## 1. Background

Crohn’s Disease (CD) is a chronic inflammatory condition that follows an unpredictable, relapsing and remitting trajectory through the course of an affected individual’s life [1]. It is believed to result from a complex interplay between genetic susceptibility, environmental factors and altered gut microbiota [1]. CD can affect all segments of the gastrointestinal tract with the most common being involvement of the terminal ileum and colon [1]. In population studies, about 25% of patients with CD develop complications of perianal fistulae within the first two decades of diagnosis [2,3,4] and perianal fistulae can be the initial manifestation of CD in about 10% of patients [5,6,7]. The age at onset of CD also appears to influence the development of perianal disease with younger patients at greatest risk.

The presence of perianal fistula in CD tends to denote an aggressive phenotype. When perianal disease is the first manifestation of CD, severe disease is likely and there may be rapid progression from inflammatory manifestations to stricturing or penetrating complications [7]. Despite improvements in medical and surgical management, pCD remains a difficult entity to treat. The refractory nature of the disease calls for significant improvements in our understanding and classification, to help streamline combined medical and surgical management.

## 2. Current Management of Perianal Fistulizing CD (pCD)

### 2.1. Classification

Classification of pCD to date has been largely limited to “simple” or “complex”, as defined by the AGA, or anatomically by the Parks’ classification [8]. A simple fistula is a superficial, intersphincteric or low trans-sphincteric fistula with only one opening, and it is neither associated with an abscess nor connected to an adjacent structure. A complex fistula is high (high intersphincteric or high transsphincteric or extra-sphincteric or suprasphincteric) and may have multiple external openings, or be associated with a perianal abscess, rectovaginal fistula, anorectal stricture or the presence of active rectal disease at the endoscopy [5,6,9]. The classification of disease burden has been guided by perianal disease activity index (PDAI) scoring, with some disease activity being captured by clinician scoring as part of the PDAI [10]. However, the scoring system can be skewed by independent variables and may not capture the global disease burden related to the perianal fistulizing disease [10].

### 2.2. Assessment

Currently, the MRI remains the gold standard imaging modality of perianal fistula [11]. Schwartz et al. [12] showed, in a small study of 32 patients, that the EAUS (endoanal ultrasound) was comparable to the MRI and when combined with examination under anesthesia, both modalities provided 100% accuracy. Another study found that the MRI was more sensitive (0.97, 95% CI 0.92–1.01) than clinical examination (0.75, 95% CI 0.65–0.86) but comparable to the EAUS (0.92, 95% CI 0.85–0.99) in differentiating a complex from a simple disease [13]. Finally, a meta-analysis comparing MRI and EAUS for a perianal fistula assessment included four studies and found comparable sensitivities for the MRI [0.87, 95% confidence interval (CI) 0.63–0.96] and the EAUS (0.87, 95% CI 0.70–0.95), although the specificity for the MRI (0.69, 95% CI 0.51–0.82) was higher than that for the EAUS (0.43, 95% CI 0.21–0.69) [14].

### 2.3. Medical Treatment

Evidence around antibiotic use in pCD is restricted to temporary symptomatic improvement only [15]. Thiopurines also have limited evidence in pCD with small studies showing only modest benefits. In the current era of biologics, thiopurines are not recommended as stand-alone therapy for fistula closure [15,16,17].

In contrast to antibiotics and thiopurines, Anti-TNF agents have achieved the best available evidence for the treatment of complex perianal CD [15]. Recent ECCO guidelines on the management of pCD recommend Anti-TNF therapy as the preferred initial treatment choice for induction medical therapy in complex perianal CD [15]. Newer biologic agents, Ustekinumab and Vedolizumab, are now in routine clinical use in luminal CD. However, their role in the management of pCD remains unclear due to the limited high quality evidence. Furthermore, with the implication of the JAK-STAT signaling pathway in patients with pCD [18] and the evolution of JAK inhibitor therapy in inflammatory bowel disease (IBD), small molecules may play a key role in the future medical management of pCD. Table 1 highlights the key evidence to date relating to biologic/small molecule therapy in pCD.

There is limited evidence around the role of de-escalation of medical therapy in the setting of good disease control in pCD with data generally suggesting an unfavorable prognosis. Mak et al. [41] reviewed consecutive patients with pCD treated with Anti-TNF therapy and subsequent discontinuation from institutes in Hong Kong, Shanghai, Taiwan, Malaysia, Thailand and Singapore. After a median follow-up of 89 months, 44 of the 78 patients (56.4%) relapsed after stopping anti-TNF therapy [41]. The cumulative probabilities of pCD relapse rates were 50.8%, 72.7% and 78.0% at 12, 36 and 60 months. Among those with relapse, however, for retreatment with anti-TNF induced remission in 24 or 29 patients (82.8%) [41], 12 patients (27.3%) required defunctioning surgery and one patient required a proctectomy. The radiological remission of pCD prior to the cessation of anti-TNF had a somewhat more favorable prognosis with only five of the 17 patients (35.3%) relapsing but with a median of 6 months [42]. A more recent meta-analysis of 309 patients from 12 studies by Huinink et al. [42] showed similar outcomes with 75/168 (45%) patients developing a relapse of pCD after the discontinuation of anti-TNF therapy after a median follow up of 11 months [42]. Among these, 58/75 (77%) patients experienced perianal fistulizing relapse and 16/75 (21%) had both perianal fistulizing relapse and anal abscess [42]. The cumulative incidences for the relapse of perianal disease were 25% at 1 year and 36% at 2 years. Overall, anti-TNF re-treatment was effective in 82% of patients [42].

#### Surgery

The surgical management of pCD most commonly consists of the drainage of sepsis with or without seton placement, and sometimes subsequent removal [43]. Clinical guidelines advocate for surgical drainage alongside the use of anti-TNF therapy in initial management [15,44], and are supported by data from retrospective studies indicating improved healing rates when compared to medical treatment alone [45,46].

Studies of reparative surgery in pCD focus largely on minimally invasive techniques that avoid the division of the sphincter complex. Rectal advancement flaps involve mobilizing a flap of tissue to occlude the internal opening of the fistula tract, preventing inflammation triggered by gastrointestinal micro-organisms. Meta-analyses have found that success and recurrence rates in pCD are around 61 and 18%, respectively [47], with a risk of continence disturbance in 9% [48]. The Ligation of the Intersphincteric Fistula Tract (LIFT) procedure has also been used in pCD and is reserved for trans-sphincteric tracts, where the tract is disrupted by dissection and ligation in the intersphincteric plane. Success rates are in the region of 53–65% with an incontinence rate of approximately 1.6% [47,49]. The recent PISA trial (Treatment of Perianal Fistulas in Crohn’s Disease, Seton Versus Anti-TNF Versus Surgical Closure Following Anti-TNF) [50] investigated rates of reinterventions when surgical repair with either LIFT or the advancement flap after anti-TNF induction was compared with chronic seton drainage or anti-TNF treatment alone. The trial was terminated early due to the high rate of reintervention required in the seton group (74% of 10 patients); however, patients undergoing surgical repair experienced the lowest rate of reinterventions (three of 14 patients, 23%) at a median of 11 months (interquartile range 10–11) post procedure [50].

Less-well-studied minimally invasive techniques take various forms, generally involving cauterizing the internal surface of the tract, or scraping and then filling it. The Video Assisted Anal Fistula Treatment (VAAFT) achieves cautery under direct vision through cannulation of the tract with a rigid fistuloscope; however, studies on patients with pCD are scarce [51]. Two studies, each involving 11 patients, demonstrated success in three (27%) and nine (82%) patients; however, both had average follow-up times of less than a year [52,53]. The technique has demonstrated better outcomes when used for symptom control, with studies finding between 46–84% of patients reporting an improvement in symptoms such as pain and discharge following (p)VAAFT [52,54]. Laser Fistula Treatment (LAFT) uses a laser fiber to cauterize the internal surface of the tract, with a recent meta-analysis of six studies involving 50 patients with pCD finding a pooled primary healing rate of 62% [55]. The use of anal fistula plugs in pCD is not recommended [15], as the rate of fistula closure has been found to be comparable to patients undergoing seton removal alone, but with increased frequency of adverse events [56]. The efficacy of fibrin glue is similarly poor (38% demonstrating remission 8 weeks post procedure) [57].

Despite (or perhaps due to) the wide range of procedures available for definitive treatment, most surgeons adopt a conservative approach [43]. This may be influenced by fears of poor wound healing and the continence risk in a population pre-disposed to stool frequency, alongside the fact that medical management avoids these complications but ostensibly achieves similarly variable yet modest rates of fistula closure [58,59], although the definition of fistula healing in most medical studies has been weak.

However, there is evidence to suggest that a combination of medical and surgical treatment results in improved healing rates over either treatment alone [60]. Hesitancy in embarking on surgical management is further perpetuated by the relative infrequency of studies investigating the efficacy of surgery. The studies that are published are constrained by small, heterogenous sample sizes, retrospective methodology, the inadequate duration of follow-up and varied, unvalidated endpoints, making it challenging to make evidence-based decisions or determine the superiority of any one treatment over another [61].

There has been a recent shift towards addressing these deficiencies in pCD research, with several multi-center RCTs being conducted to examine the efficacy of various surgical and medical treatments. In doing so, these trials have revealed several important issues to consider in optimizing patient management and in undertaking pCD research.

The previously mentioned PISA Trial [50] aimed to compare surgical and medical management of pCD by randomizing patients to treatment groups of chronic seton drainage, anti-TNF treatment or surgical repair with either LIFT or an advancement flap post anti-TNF induction if they had no treatment preference, or, latterly, enrolled them into a prospective registry if treatment preference was a reason to decline participation. Both groups were managed according to the same protocol and demonstrated similar levels of disease severity; however, the number of reinterventions required in the patients randomized to chronic seton drainage was significantly higher than those receiving anti-TNF with or without surgical closure, leading to early termination. This finding was not replicated in the prospective registry patients, the only difference being that those in the registry actively chose seton drainage rather than being randomized to receive the treatment, which the authors suggest may have meant that treatment was better tolerated, and therefore, influenced the primary endpoint of re-intervention [50]. Patients who choose seton drainage may do so in order to avoid the side effects and complications of other modalities—such as the loss of security seen after seton removal, with the attendant risk of abscess formation—or may have a treatment goal of clinical stability rather than fistula closure. Whilst the trial data was unable to indicate the optimal treatment strategy, it highlighted the importance of establishing patient priorities in pCD to guide decision-making regarding the most suitable treatments, and to individualize the definition of treatment ‘success’. It is, therefore, essential that any future management strategy has patient-centered decision-making at its core.

With this in mind, a continuation study (PISA-II) investigated clinical and radiological outcomes for patients undergoing anti-TNF treatment and seton removal with those receiving short-term anti-TNF treatment combined with surgical closure (seton removal followed by advancement flap or LIFT) in a patient preference randomized study. Although there were no differences in the rates of clinical fistula closure, radiological healing, defined as a completely fibrotic tract or tract with a MAGNIFI-CD score of 0, was significantly higher in the surgical closure group (32% vs. 9% in the anti-TNF group, *p* = 0.005), with lower PDAI scores and fewer reinterventions than the anti-TNF group at the 18-month follow up [62]. The importance of radiological healing in pCD has been previously established, with several studies demonstrating improved long-term outcomes when deep tissue healing is demonstrated on MRI [63,64]. However, PISA II showed that surgical treatment alongside anti-TNF therapy is more likely to result in deep tissue healing and, therefore, durable fistula closure than medical treatment alone, further promoting the benefits of combined medical and surgical treatment, and also that radiological healing is a valuable independent outcome, as it correlates with improved quality of life in the short term, and avoidance of recurrence in the long term [62].

The benefits of surgical closure were similarly highlighted by the ADMIRE CD study [65], where the primary aim was to investigate the efficacy of Mesenchymal Stem Cells (MSCs) to treat pCD refractory to the gold standard medical treatment. Whilst the success of MSCs in this study is well known, it is the finding that 36% of the comparator arm (consisting of a group that received similar treatment but for an injection of normal saline in place of MSCs) demonstrated clinical remission at 24-weeks post-treatment that holds additional significance, as it supports the idea that a proactive surgical approach, aimed at addressing the anatomical factors influencing fistula persistence in pCD, is required to achieve closure in a select group of patients [65]. The longer-term follow up data of 24 patients who initially showed clinical remission shows that closure persists in just over half at 156 weeks post-treatment [66], emphasizing that the management of pCD should extend beyond the traditional, low-risk (and probably lower yield than previously thought) option of seton removal alone if lasting fistula closure is to be achieved.

## 3. The Future of Treating Perianal CD

### 3.1. Better Classification of Perianal CD

**Figure 1:**
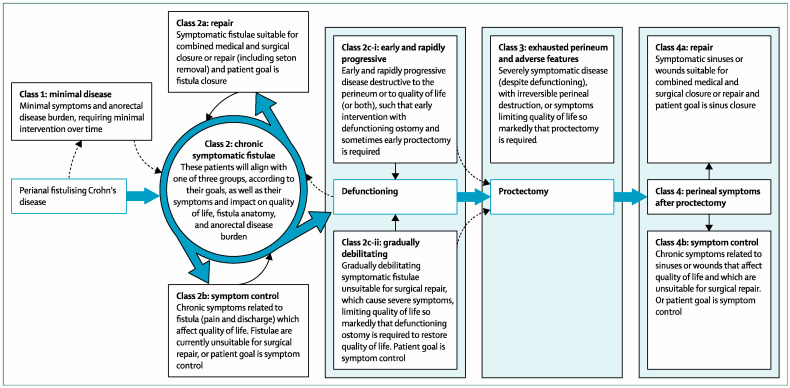
Perianal CD Classification (Reprinted with permission from Lancet Gastroenterology and Hepatology) [67].

We believe the future of pCD management begins with the reclassification of the disease process to incorporate patient goals along with the anorectal disease burden to streamline medical and surgical management and improve the quality of research. An international consortium of expert centers [67] has recently published a comprehensive classification (Figure 1) which enables patients with pCD to be stratified into various classes according to disease severity as well as disease outcome; the synchronization of patient and clinician goals in decision making, with a proactive, combined medical and surgical approach, on a treat-to-patient goal basis; and the identification of indications for curative fistula treatment, diverting ostomy and proctectomy [67]. This classification crucially centers management around patients’ goals, based on the significant impact of disease on a patient’s quality of life, and the PISA study’s findings [67].

Future studies and clinical management may be guided by this classification, which will enable the stratification of patients within an appropriate treatment algorithm but will also encourage researchers to select and stratify patients more effectively in clinical trials.

### 3.2. Better Understanding of the Pathophysiology of Perianal CD

There is an urgent need to improve our understanding of the immunobiology of pCD, to uncover novel drivers of disease and to unmask new medical therapies. Two key immunopathogenic mechanisms, the epithelial-to-mesenchymal transition (EMT) and extracellular matrix (ECM) degradation, are thought to be involved in pCD. EMT is a process of transformation from a differentiated epithelial cell to a mesenchymal cell through an intermediary (“transitional cells”), whereby they become more migratory with increased cell spreading and upregulation of mesenchymal proteins such as Vimentin [7,68]. This process is associated with embryogenesis, organ development and wound repair but is also involved in pathological fibrosis, tumor growth and metastasis [7,68]. In pCD, fistulae are lined wholly or partly by myofibroblast-like cells/transitional cells (TCs) that have lost expression of the epithelial cell–cell adhesion protein E-cadherin (*CHD1*) and upregulated expression of the cell invasion marker integrin-β6 (*ITGB6*), but retain the expression of cytokeratin-8 and -20, likely representing intestinal epithelial cells that have undergone epithelial-to-mesenchymal transition (EMT) [69]. Several transcription factors known to regulate EMT, such as SNAIL (*SNAI1*) and to a lesser extent SLUG (*SNAI2*), have been found to be expressed in TCs that line the fistula tract, as well as other EMT-associated transcription factors like Protein-C-ets-1 (*ETS1*) and integrin-β6 [69,70,71]. EMT is further facilitated by the production of matrix metalloproteinases (MMPs) that degrade the surrounding ECM, allowing the penetration of cells undergoing EMT, now with enhanced migratory and invasive potential, into adjacent tissues. MMPs two, three and nine have been shown to be expressed in perianal fistulas, whereas their natural inhibitors, tissue inhibitors of matrix metallaoproteinases (TIMPs), were found to be undetectable, suggesting that a dysregulation of the MMP-inhibitor relationship may be an important feature of ECM remodeling in pCD [72,73]. Rizzo et al. recently discovered that once the dysfunctional ECM is established, mechanical properties and composition of ECM drive EMT [74]. In turn, EMT sustains changes in the ECM components through the TNF-stimulated gene-6 (TSG-6), pathological HA-HC complexes (covalent complexes between hyaluronan and the heavy chains (HCs) of inter-alpha- inhibitor (IaI)), thus creating a feedback between ECM remodeling and EMT processes [74].

However, the potential triggers of EMT in pCD are not well understood. Whilst bacteria are proposed as a key stimulus underlying the aberrant immune-environment in pCD [75], our understanding of how microbes and the colonic microbiota specifically interact in fistula pathogenesis is limited. Perineal infection has been suggested as the initial insult triggering fistula formation [76]; yet, established fistula tracts are generally devoid of bacteria on 16S rRNA sequencing and fluorescent in situ hybridization [77,78]. Pro-inflammatory peptidoglycan is, however, isolated in 90% of fistulae and has been linked to a reactive immune response [78,79], suggesting that remnant bacterial antigens may participate in fistula development or persistence following clearance of living microbes. Bacterial endotoxins may also interact with, and enter, fistula tracts at the internal opening. Microbial interactions here seem likely given the colostomy, and diversion of the fecal stream, is sometimes beneficial in fistula healing and inflammation [79]. Reduced goblet cell predominance in the anorectum of fistula patients, compared to healthy controls, has been noted and may potentiate susceptibility to microbial-induced cytokine activation [79]. Furthermore, differences have been noted in the microbiota of patients with pCD compared to luminal CD [79,80,81,82]. Developing our understanding of bacterial interactions with fistulae, and identifying other potential participants (i.e., viral and mycotic factors), is a key area for research and may give new avenues for future therapies.

Finally, genetic studies shall undoubtedly continue to yield pathophysiological insights in the area. Alongside highlighting the JAK-STAT pathway, genotyping has identified specific variations in genes relating to the host–pathogen response (i.e., NOD2 s72796353, IBD5 and NCF4) [83,84,85,86], autophagy (IRGM) [87,88], cytokine production (IL-10 and TNFSF15) [89,90], the epithelial barrier (DLG5) [91] and lymphocyte homing (NKX2-3) [90], all which predispose to pCD. The function of many associated genes is unclear. For instance, CDKAL1 is associated with pCD, despite its exact role being unknown [92]. Yet, CDKAL1 transcripts are highly expressed by CD4+ lymphocytes [93]. Conversely, the genetic factor PUS10, involved in RNA signaling regulation [94], was protective against pCD in a large European cohort [95]. Again, the mechanism of this effect is obscure. Such findings provide opportunities to explore new targets for fistula susceptibility and protection. Large datasets are still needed for meaningful genetic comparisons in pCD. However, as evidence grows, it is easy to see genetics providing further clues to all aspects of fistula pathogenesis, from immunology and microbiological response to tissue healing and ECM integrity.

**Figure 2:**
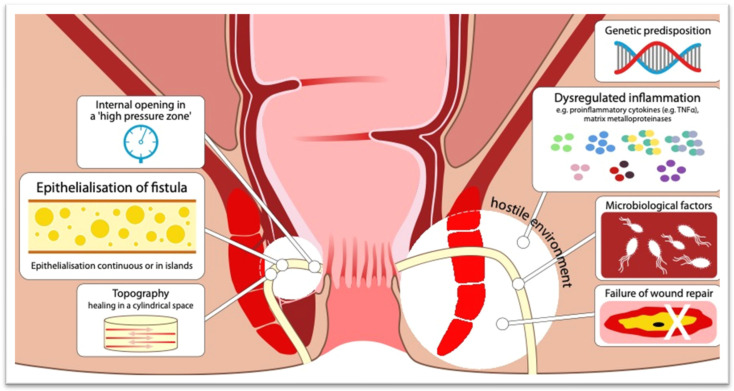
Key factors that promote or facilitate perianal fistulization in CD. The dysregulated inflammation cascade hallmarking the ‘hostile environment’ is further explained in Figure 3 below.

**Figure 3:**
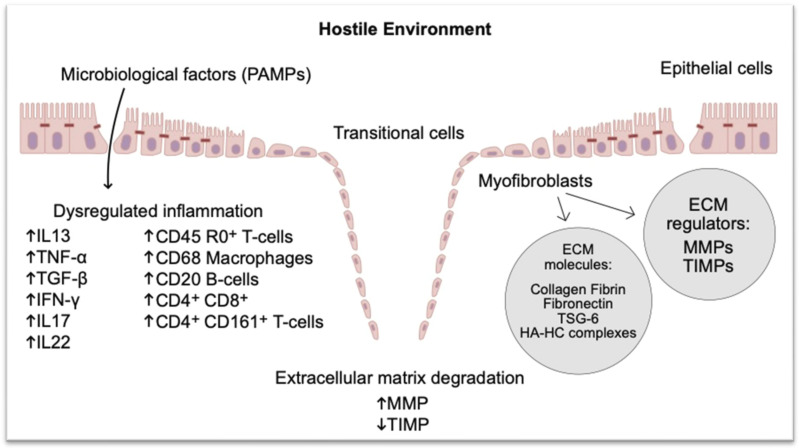
Pathogenesis of Crohn’s perianal fistula. Pathogenesis of Crohn’s perianal fistula. Pathogen-associated molecular patterns (PAMPs) such as Muramyl dipeptide (MDP) lead to activation of various immune cells and cytokine release causing a dysreglated inflammatory cascade resulting in epithelial-to-mesenchymal transition. Subsequent activation of myofibroblasts leads to increased expression of matrix metalloproteinases (MMP) and decreased expression of tissue inhibitors of MMPs (TIMP) resulting in extracellular matrix degradation and failure of wound repair. TSG-6: TNF-stimulated gene-6; HA-HC complexes: covalent complexes between hyaluronan and the heavy chains (HCs) of inter-alpha-inhibitor (lal).

Therefore, the current proposed theory to describe the immunopathogenesis of pCD suggests an aberrant immune response to bacterial stimuli in a genetically susceptible individual, leading to a dysregulated inflammatory response characterized by secretion of pathogenic cytokines like TNF-α, IL-13 and TGF-β, all of which have been shown to be abundantly expressed in perianal fistulas (Figure 2 and Figure 3). TGF-β has been found to induce higher expression of SNAIL in colonic lamnia propria fibroblasts (CLPFs) isolated from patients with fistulizing CD, as well as in in vitro spheroid models of EMT [68]. TGFβ is also capable of inducing IL13 production in CLPFs, more so in patients with fistulizing disease versus non-fistulizing CD, which can itself induce expression of EMT-associated transcription factors and cell invasion markers like SLUG and β6-integrin, suggesting the two cytokines act synergistically to induce EMT [68]. Similarly, TNFα has been shown to induce expression of ETS1 and integrin-β6 in CLPFs, an effect that was reversed upon the administration of anti-TNF in vitro [71]. Clearly, cytokines are important inducers of EMT, enhancing cell invasiveness and migratory potential, resulting in the destruction of the basement membrane and ECM and furthering the spread of new mesenchymal-like cells into deeper layers of tissue.

The cytokine-mediated control of EMT clearly implicates dysregulated immune responses as a central factor in the pathogenesis of pCD. Efforts have been made to characterize the immune environment in perianal fistulas. CD45RO^+^ CD3^+^ memory T-cells have been observed in the interior wall of the fistula tract in patients with pCD, predominantly CD4^+^ and CD8^+^ T-cells [96,97]. Additionally, an accumulation of IFNγ and IL17 producing CD161^+^ CD4^+^ T-cells displaying a Th1, Th1-Th17 hybrid and Th17 phenotype have been observed in fistula curettage tissue compared to peripheral blood of patients with pCD [98], implying that T-cells are a potential important participant in fistula pathogenesis. This is further evidenced by the fact TNFα production by PBMCs in patients with pCD has been shown to be predominantly derived from CD3^+^ T-cells. When epithelial cells are exposed to it in vitro, it induces the expression of *SNAI2* and *ETS1*, an effect interestingly potentiated by the co-administration of IL22 [97]. Limited data are available in relation to the phenotype of other cellular compartments in pCD. CD68^+^ macrophages and CD20^+^ B-cells have been detected in the lining of the fistula tract [96], and the phenotypic analysis of cells isolated from fistula tissue in a small cohort of six patients with pCD showed that the majority of cells in fistulas were of myeloid origin, displaying a CD11b^+^ CD11c^+^ DC-like phenotype [99]. Additionally, as part of the same study van Unen et al. [99] also identified the presence of CCR6^−^ ILC3s in fistula tissue.

### 3.3. Improved Management May Need All Aspects of Pathogenesis to Be Addressed—Combining Therapies

Finally, the complex interplay of ECM remodeling and EMT processes in pCD immunopathogenesis suggests that no single treatment can successfully manage fistula healing [74]. Local mesenchymal stem cell (MSC) injection is perhaps the most promising treatment on the horizon for patients with pCD [100]. The body of evidence for their utility has been growing ever since the first phase I clinical trial in 2005 in which Garcia-Olmo et al. showed up to a 60% healing rate with the use of adipose-derived MSCs [101]. A subsequent larger Phase II trial performed on patients with Crohn’s and idiopathic fistulizing disease demonstrated healing in 46% of patients following a single-dose administration of 20 million autologous MSCs in combination with fibrin glue treatment. This success rate further increased to 71% in those who received a second dose (40 million MSCs) if fistula healing was not seen at week 8, striking results compared to the 16% of patients who experienced fistula healing with fibrin glue treatment alone [102]. In the largest of these, the Phase III multi-center ADMIRE-CD study, 212 patients with treatment refractory pCD underwent EUA with fistula tract curettage and surgical closure of their internal opening [65]. They were treated with two operations including closure of the internal opening, and randomized to placebo or a single injection of darvadstrocel (120 million expanded human allogeneic adipose-derived MSCs). Combined radiological and clinical remission, the latter defined as closure of external openings and absence of fistula drainage, was seen in 56% of stem cell patients at 52 weeks (compared to 39% of controls) [65]. Extended follow-up suggested remission rates extend beyond 104 weeks, with no safety concerns seen [66]. In addition, a recent stem cell therapy meta-analysis and systematic review of 29 articles including 1252 patients found the stem cell group had a higher rate of fistula healing compared to the placebo group in patients of Crohn’s fistula (62% vs. 40%, OR 2.21, 95% CI 1.19 to 4.11, *p* < 0.05) [103]. The currently running ADMIRE-II multicenter study should shed more light on their efficacy.

The positive results of MSCs are likely due to their multifaceted therapeutic action targeting key pCD pathogenic factors such as inhibiting the dysregulated inflammation leading to EMT and ECM degradation and assisting in wound healing along with its combined surgical delivery approach of internal opening closure which leads to disconnection of the tract from the gut [104,105]. MSCs respond to tissue inflammation by modulating the local innate and adaptive immune system in favor of a regulatory, tissue-healing, environment [106,107]. This is achieved through direct cell-to-cell signaling alongside paracrine release of molecules including regulatory cytokines, chemokines, growth factors and pro-angiogenic molecules [106,107]. Specifically, they inhibit inflammatory dendritic cell and CD4+ cell activity whilst promoting the action of regulatory T-cells [106,107,108].

This ‘biological’ effect has been paired in most studies with only rudimentary surgical repair (such as the ‘scrape and close’ in the ADMIRE trial) in order to maximize the impact of the investigational product within the trial, and perhaps to remove some of the heterogeneity seen in surgical repair. An interesting question so far unasked is whether MSCs ‘augments’ the success of the best surgical repairs, currently thought to be LIFT and flap, in patients whose fistulae are suitable for anatomical repair. An augmentation study like this might not demonstrate a substantial benefit of MSCs over a high quality repair, but if it did, it would represent a new gold standard of care, with medically optimized patients, treated with the optimal surgical strategy augmented by a local biological agent. The authors contend that this is the treatment they would like for their own Class 2a fistulae.

Current work on MSCs have proven efficacy and given an understanding of optimal cell dosage [103]. Whilst there are still delivery questions, such as the benefit of repeat injections in non-responders, the main challenge is how we can bring this treatment into everyday care. More evidence is needed before we can hope to fund cellular therapy in most healthcare systems. Additionally, proper infrastructure is needed to prepare and deliver MSCs on a scale to universally treat refractory pCD [109,110]. Despite the evolving evidence to date, it remains unclear where MSCs fit into the pCD management algorithm; should they be used early or late in the disease process, how can we surgically and medically optimize patients prior to receiving stem cell therapy and importantly, are we able to identify clinical, biochemical or immunological predictors of treatment success? Studies of MSC action in other autoimmune disorders [111] and preliminary results from studies of pCD [112] have laid the groundwork in which to do this, but larger studies are required to develop robust conclusions. As the evidence base evolves on stem cells in pCD, we believe their role in management can be defined into the various classification classes mentioned above, in order to support their role in the management of pCD.

As scientific understanding of stem cells progresses, already we have seen the next step in this field. Extracellular vesicles (EVs), released by MSCs, hold the active paracrine molecules driving the majority of beneficial effects the cells have in tissue [104]. Through culturing MSCs in the laboratory, EVs can be isolated and injected in the place of MSCs. Recent studies have suggested EVs could have comparable effects to MSCs for fistula patients [109,113]. Utilizing EVs is likely to improve costs and other difficulties, such as transport and storage requirements for living cells, that prove challenges for MSC use [109,113]. By adapting MSCs from a cellular to molecular therapy, EVs could overcome barriers preventing this treatment coming into routine practice.

Furthermore, MSCs’ benefit over conventional drug-based immunological therapies in affecting multiple pathogenic mechanisms of fistula formation raises the question of how else combining medical and timely surgical care could lead to better outcomes in perianal CD. The role of combined biologic therapy in IBD is evolving with several case series reported to date with the largest one to date containing only 22 patients with 24 therapeutic trials of dual biologic therapy (DBT) [114]. Of these, 11 therapeutic trials (50%) had active perianal fistulas at the time of DBT and among these, the presence of perianal fistulas declined from 50% at the baseline to 33% post-treatment [114]. The early promise of JAK inhibition and Ustekinumab in pCD to date, along with the well-established benefits of Anti-TNF therapies, may propose a role for combination therapy in those patients with an early aggressive disease such as those in Class 2c-i or those with progressive debilitating disease such as the Class 2c-ii patients. Key questions that remain unanswered include assessing the exact role and safety of combination biologics in pCD, using surgical drainage to help recapture biologics’ efficacy and identifying early clinical, radiological and immunological predictors of treatment success.

Finally, as part of combined and collaborative management algorithm of pCD, we believe it is pivotal to address and treat the co-existing psychological morbidity associated with the disease. Studies have shown that 73% of patients self-reported periods of depression and 13% suicidal ideation whilst living with pCD along with sexual function and body image also being affected [115,116,117]. Therefore, it is crucial that psychological wellbeing is regularly screened for and promptly addressed when managing these patients.

## 4. Conclusions

In this article, we have discussed our current understanding of the immunopathogenesis of pCD along with evidence relating to the various medical and surgical management options available to clinicians managing patients with pCD. However, despite the evolving medical and surgical therapy options, evidence around them is somewhat limited by the retrospective nature of most studies and there appears to be a therapeutic ceiling with treatment success in pCD. Furthermore, mesenchymal stem cell therapy has shown promise in early Phase 3 studies and may present a new era of managing pCD. Nonetheless, due to its refractory phenotype, the key to improving pCD management may lie in combining multiple therapies such as MSCs with specific medical and/or surgical therapies. We believe to embark on this era of streamlining optimized pCD management; we also need to improve our understanding of the immunology of pCD, our classification of the disease process and identification of early predictors of treatment success.

## Figures and Tables

**Table 1:** Evidence of biologic and small molecule therapy in pCD.

Therapy	Evidence to Date in pCD
Anti-TNF	Present et al. showed 50% reduction in fistula drainage in 68% of patients in the Infliximab 5 mg/kg induction dose cohort compared to 26% of patients treated with the placebo (*p* = 0.002) [19]. The ACCENT II trial then went on to show that maintenance IFX for a period of 54 weeks was superior to the placebo in patients who responded to induction therapy [20]. At week 54, 36% of patients in the IFX maintenance group had a complete absence of draining fistulas compared with 19% in the placebo group (*p* = 0.009) [20]. Furthermore, multiple retrospective studies have assessed the benefit of Infliximab including higher serum levels (ranging from >7.2 to >20) [21,22,23] corresponding to improved clinical and radiological remission outcomes [21,22,23,24,25,26,27]. In the subgroup analysis of the CHARM study, fistula healing was seen in approximately 60% of patients after 2 years of Adalimumab therapy [28]. In the CHOICE trial, 39% of patients who had complete fistula healing to Adalimumab therapy were primary or secondary non-responders to Infliximab [29]. However, the ACCESS study suggested that fistula healing rates in Adalimumab-treated patients were much higher for Infliximab naïve patients than Infliximab-experienced (60% versus 28%, respectively; *p* < 0.01) [30]. Similar to studies of Infliximab in pCD, higher Adalimumab serum levels correlated with improved fistula outcomes [26,27]. Furthermore, local intra-fistula Anti-TNF injections have also been trialled in small pilot studies with mixed results and a lack of long-term data [31].
Ustekinumab	A subgroup analysis of the pivotal studies of Ustekinumab in CD (UNITI-1, UNITI-2 and CERTIFI) showed complete fistula resolution in 25% of all pooled Ustekinumab patients at week 8 compared to only 14% of the pooled placebo group patients with active fistula (*p* = 0.073) [32]. In addition, a post-hoc pooled of the STARDUST and SEAVUE studies showed an overall 50% of patients receiving Ustekinumab had clinical fistula healing at the end of the study with no impact of trough levels or dosing intervals seen in the small numbers of patients studied (17 and 12, respectively) [33]. A Dutch nationwide prospective observational cohort study of 28 patients showed 36% complete clinical resolution after 24 weeks of treatment in patients who had prior anti-TNF exposure [34]. The BioLAP multicenter study from the GETAID group showed clinical success at 6 months (as assessed by the physician’s judgment without additional medical or surgical treatment) occurred in 38.5% (57/148) of patients. In this study, 33% of patients (29/88) with setons at Ustekinumab initiation had successful removal [35]. Finally, a recent retrospective analysis showed, in patients who had received at least 16 weeks of Ustekinumab therapy, clinical remission and response rates were 40.7% and 63.0%, respectively. The study also went on to show radiological healing observed in 44.8% [35]. An Ustekinumab trough concentration over 2.11 μg/mL was correlated with a higher likelihood of perianal fistula clinical remission [36].
Vedolizumab	The GETAID BioLAP study also assessed 102 patients with active perianal disease treated with Vedolizumab, in whom success was reached in 23 patients (23%). Among patients with setons at initiation, 9/61 (15%) had a successful removal [37]. The ENTERPRISE study was a randomized trial evaluating the effectiveness of two different 22-week Vedolizumab treatment regimens in pCD with 78.6% of the patients having had previous Anti-TNF exposure [38]. Unfortunately, the study was ceased prematurely due to recruitment challenges resulting in low patient counts and as a result, all analyses were descriptive. In the standard dosing group, 9/14 patients (64%) showed ≥50% reduction from the baseline in the number of draining perianal fistulae at week 30 [38]. In contrast, only 6/14 patients (43%) in the group that received an additional week 10 dose achieved ≥50% reduction in drainage [38].
Small molecules	Filgotinib, a selective JAK1 inhibitor, has shown good efficacy in pCD in the Phase II DIVERGENCE 2 study. The proportion of patients who achieved a combined fistula response at week 24 was numerically higher in the FIL 200 mg group (47.1%; 90% confidence interval [CI]: 26.0–68.9) than in the PBO group (25.0%; 90% CI: 7.2–52.7) [39]. Upadacitinib (UPA), another selective JAK1 inhibitor, has shown some promise in its pivotal studies. The subgroup analysis of U-EXCEL, U-EXCEED and U-ENDURE studies showed that 124 patients had perianal fistulas. Of these, the proportion of patients who achieved the complete resolution of draining and ≥50% reduction in draining was higher with UPA vs placebo at week 12 (47.7% vs. 9.1%; *p* = 0.002 and 50.0% vs. 13.6%; *p* = 0.004). However, the response was not statistically significant at week 52 [40].

## Data Availability

The authors confirm that the data supporting the findings of this review is cited in both the text and the reference list.
